# Phenotypic Investigation and Detection of Biofilm-Associated Genes in *Acinetobacter baumannii* Isolates, Obtained from Companion Animals

**DOI:** 10.3390/tropicalmed9050109

**Published:** 2024-05-11

**Authors:** Marios Lysitsas, Eleutherios Triantafillou, Irene Chatzipanagiotidou, Konstantina Antoniou, Vassiliki Spyrou, Charalambos Billinis, George Valiakos

**Affiliations:** 1 Faculty of Veterinary Science, University of Thessaly, 43100 Karditsa, Greece; mlysitsas@uth.gr (M.L.); billinis@uth.gr (C.B.); 2 Vet Analyseis, Private Diagnostic Laboratory, 41335 Larissa, Greece; eltriantafil@gmail.com (E.T.); antonioukwn@gmail.com (K.A.); 3 Department of Biochemistry and Biotechnology, University of Thessaly, 41500 Larissa, Greece; eichatzip@uth.gr; 4 Department of Animal Science, University of Thessaly, 41334 Larissa, Greece; vasilikispyrou@uth.gr

**Keywords:** *Acinetobacter baumannii*, biofilm, companion animals, antibiotic resistance, *bap*, *bla*_PER-1_, *csu*E, *omp*A

## Abstract

Bacteria of the genus *Acinetobacter*, especially *Acinetobacter baumannii* (*Ab*), have emerged as pathogens of companion animals during the last two decades and are commonly associated with hospitalization and multidrug resistance. A critical factor for the distribution of relevant strains in healthcare facilities, including veterinary facilities, is their adherence to both biotic and abiotic surfaces and the production of biofilms. A group of 41 *A. baumannii* isolates obtained from canine and feline clinical samples in Greece was subjected to phenotypic investigation of their ability to produce biofilms using the tissue culture plate (TCP) method. All of them (100%) produced biofilms, while 23 isolates (56.1%) were classified as strong producers, 11 (26.8%) as moderate producers, and 7 (17.1%) as weak producers. A correlation between the MDR and XDR phenotypes and weak or moderate biofilm production was identified. Moreover, the presence of four biofilm-associated genes *bap*, *bla*_PER_, *omp*A, and csuE was examined by PCR, and they were detected in 100%, 65.9%, 97.6%, and 95.1% of the strains respectively. All isolates carried at least two of the investigated genes, whereas most of the strong biofilm producers carried all four genes. In conclusion, the spread and persistence of biofilm-producing *Ab* strains in veterinary facilities is a matter of concern, since they are regularly obtained from infected animals, indicating their potential as challenging pathogens for veterinarians due to multidrug resistance and tolerance in conventional eradication measures. Furthermore, considering that companion animals can act as reservoirs of relevant strains, public health concerns emerge.

## 1. Introduction

Members of the *Acinetobacter baumannii–calcoaceticus* (*Abc*) complex, especially *Acinetobacter baumannii*, are troublesome pathogens in human medicine, as they have constituted a major cause of nosocomial infections during the last decades and are associated with high mortality rates and multidrug resistance [1]. Their distribution and persistence in the hospital environment are strongly related to the ability of these species to adhere to biotic and abiotic surfaces and form biofilms [2]. Furthermore, biofilms are not only associated with survival and spreading in healthcare facilities, but also contribute to bacterial virulence and even complicate the outcome of the treatment [3].

Biofilm formation is a complex process that is affected by numerous factors, such as the production of aggregation substances, adhesion of collagen, pili expression, and iron acquisition [2]. Therefore, several genes and proteins are involved in the various developmental stages. In particular, *A. baumannii* biofilm-associated protein (bap) is essential for complete biofilm production on surfaces such as polypropylene, polystyrene, and titanium [4]. Moreover, the presence of the outer membrane protein A (ompA) enhances its attachment to both abiotic materials and eukaryotic cells, while contributing to the intrinsic resistance of this species to numerous antibiotics [5,6]. Both biofilm formation and the initial adherence of *A. baumannii* to epithelial cells are also mediated by csu pili [7,8,9]. Finally, the presence and expression of the *bla*_PER_ gene is correlated with an increase in virulence, antibiotic resistance, and development of biofilms [2,10].

In human medicine, *Ab* is frequently implicated in hospital outbreaks, associated with critically ill patients and high mortality rates, and thus, it is unsurprisingly included in the ESKAPE group of antibiotic-resistant pathogens (*Enterococcus faecium*, *Staphylococcus aureus*, *Klebsiella pneumoniae*, *Acinetobacter baumannii*, *Pseudomonas aeruginosa*, and *Enterobacter* spp.) [11]. Transmission occurs through closeness to infected patients or contaminated surfaces and medical devices, especially in intensive care units (ICUs) [11,12]. Treatment is often challenging, due to drug resistance, and regularly requires last-resort agents, combination therapies, or alternative pharmacodynamic strategies [12].

In veterinary medicine, the emergence of *Abc* strains as nosocomial pathogens, particularly in companion animals, has been documented in recent years [13]. Since 2011, outbreaks in some European veterinary clinics have been documented [14,15], while the danger that animals could act as a reservoir of multidrug-resistant (MDR) or extensively drug-resistant (XDR) strains in the community is considerable [11,16].

The objective of this study was to investigate the phenotypic and molecular aspects of biofilm formation by *Acinetobacter baumannii* isolates obtained from clinical samples of companion animals in Greece. Additionally, it indicates the emergence of *Acinetobacter baumannii* as a challenging pathogen for pets, due to the multidrug-resistant profiles of the strains and their persistence in healthcare facilities. Finally, we highlight the importance of surveillance and eradication measures to prevent further distribution of relevant isolates in the community.

## 2. Materials and Methods

### 2.1. Isolates Included in the Study

*Acinetobacter baumannii* isolates were obtained from cases of canine and feline infections in Greece between October 2020 and May 2023. Isolation, identification, phenotypic characteristics, and resistance profiles of the bacteria were described previously [17], determined by the Kirby–Bauer method and subsequently confirmed by MIC method (VITEK). The strains were maintained in Brain Heart Infusion (BHI) broth supplemented with 20% glycerol at −80 °C. Before performing the phenotypic assays, pure culture of each strain was accomplished by inoculation on a general-purpose culture medium (Tryptic Soy Agar) and incubated for 16 h at 35 °C.

### 2.2. Phenotypic Quantification of Biofilm Production by the Microplate Adhesion Technique

The polystyrene tissue culture plate method was used to quantify biofilm production by the selected isolates. The method was performed as previously described [18,19,20] with a few modifications. A quantity from a single colony of each strain’s pure culture was obtained with a 1 μL loop and inoculated in 0.9% normal saline. Then, the suspension was vortexed and its turbidity was adjusted at 0.5 McFarland, by addition of more saline or bacteria, using a turbidimeter (Standard DensiCHEK™ Plus, Biomerieux Inc., Craponne, France). Subsequently, 50 μL of this suspension was added in tubes containing 5 mL of BHI broth. The tubes were then incubated for 12 h at 35 °C. Τwo tubes of sterile BHI broth were also incubated as negative controls. Two 96-well flat-bottomed sterile polystyrene plates were used. A total of 180 μL of sterile BHI Broth supplemented with 1.1% glucose (to achieve a final concentration of 1% after inoculation of the overnight cultures) was added to each well. The first three wells of each plate were inoculated with 20 μL of negative control. Subsequently, 20 μL of each overnight culture was inoculated into three consecutive wells, according to the order of the isolate codes (Table S1). The plates were covered and incubated at 35 °C for 24 h. At the end of the incubation period, the wells were emptied, and phosphate-buffered saline (PBS) was used to wash all wells three consecutive times and remove any free-floating bacteria. Afterward, the adherent bacteria in the wells were fixed with 99% methanol (200 μL per well for 15 min). The plates were emptied and left to dry for 24 h.

A quantity of 150 μL of crystal violet 2% was added to each well to stain the adherent bacteria. After 15 min, the plates were washed carefully with tap water (until the washing was completely free of stain) and air dried. Resolubilization of the dye was performed with 150 μL of 95% ethanol per well for approximately 20 min without shaking the plates. The plates were then covered to prevent evaporation. Subsequently, the optical density (OD) of each well was measured at 595 nm using a Multiskan™ FC microplate reader (Thermo Fisher Scientific, Waltham, MA, USA).

A previously proposed classification method was evaluated to interpret the results [18,19]. Specifically, the cutoff absorbance value (ODc) was established. This was defined as the sum of the mean absorbance of the wells used as a negative control and three times the standard deviation of these controls (ODc = mean OD_NC_ + 3SD_NC_). According to this categorization, the isolates were divided into four groups by calculating the mean OD of the wells inoculated with each one of them and evaluating the classification criteria, which are presented in Table 1.

### 2.3. Detection of Biofilm-Related Genes

Molecular investigations of the four biofilm-related genes were performed. In particular, the presence of *bap*, *bla*_PER-1_, *csu*E, and *omp*A genes was examined by PCR using previously described sets of primers [2], which are available in Table 2.

Whole-genomic DNA was extracted from each strain using a commercial kit (IndiSpin Pathogen Kit, INDICAL BIOSCIENCE, GmbH). All procedures were performed according to the manufacturer’s instructions.

For each reaction, a 25 μL mix was created for each strain by adding the following reagents: 12.5 μL of Xpert Fast Mastermix (2X) with dye (GRiSP Research Solutions, Porto, Portugal), 2 μL (10 pmol) of each primer, 0.5 μL of bacterial DNA, and 8 μL of PCR-grade water. Briefly, the reaction conditions were as follows: 95 °C for 1 min, followed by 40 cycles of 95 °C for 15 s (denaturation), 60 °C for 15 s (annealing), and 72 °C for 3 s (elongation), with a final extension at 72 °C for 3 min. The DNA products were identified by electrophoresis in 0.5 Tris-borate-EDTA using 1.5% agarose gel stained with ethidium bromide solution. Positive and negative controls were included in all PCR assays.

## 3. Results

### 3.1. Included Strains, Type of Samples, and Antibiotic Resistance Patterns

A group of 41 *Acinetobacter baumannii* isolates was identified and tested for antibiotic resistance in a previous study [17]. Data regarding the origin and type of samples and the resistance profile of each strain are presented in Table S1.

### 3.2. Phenotypic Quantification of Biofilm Production

**Figure 1:**
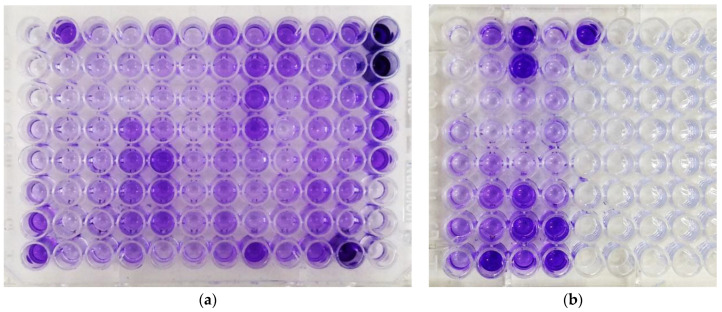
Tissue culture plate (TCP) assay: (**a**) Plate 1: Negative Control and A1 to A31; (**b**) Plate 2: Negative Control and A32 to A41.

All isolates (41/41, 100.0%) were positive for biofilm production in the TCP test (Figure 1). In particular, 23 (56.1%) were identified as strong biofilm producers, 11 as moderate (26.8%), and 7 as weak (17.1%), according to the classification criteria of Table 1 (ODc = 0.110). The mean absorbance was approximately 0.607. The detailed data are presented in Table 3 and Table S1.

### 3.3. Association of Biofilm Quantification, Sample Type, and Antibiotic Resistance

Data regarding the association between the isolate’s origin (canine of feline, sample type) and the classification of biofilm production are presented in Table 4.

Strong biofilm production was detected in the majority of the isolates of both canine and feline origin. In reference to the sample site, a distribution was documented in strains from soft tissue and urinary tract infections (UTIs), while all bacteria of other origins (otitis, respiratory system, and bacteriaemia) were classified as strong producers. Interestingly, almost all isolates (19/20) obtained from veterinary clinics located in the area of Athens were classified as strong producers, in contrast to those from the area of Thessaloniki, which were mostly classified as weak or moderate (Table S1).

Relevant data regarding the resistance rates of the strains to the antibiotics are available in Table 5.

All isolates included in this study were biofilm-producing. Nevertheless, the data included in Table 5 clearly indicate that susceptible bacteria tended to form stronger biofilms when tested by the TCP method, compared to the MDR or XDR strains, as identified previously [16]. In addition, the mean absorbance values of non-resistant isolates were over two-fold those of the resistant isolates for almost all antibiotics (Table 6). Therefore, a correlation was highlighted between multidrug resistance and weak or moderate biofilm production in the group of the investigated strains.

### 3.4. Detection of Biofilm-Related Genes

**Figure 2:**
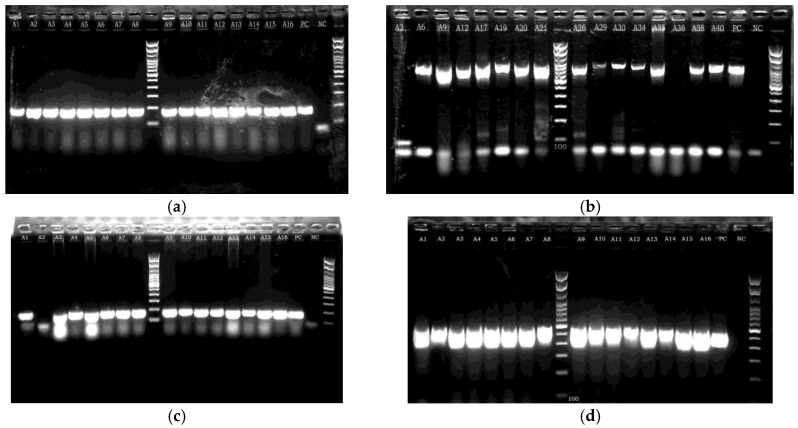
Indicative PCR gel electrophoresis images: (**a**) product of approximately 184 bp size detected in isolates A1 to A16, indicative of the presence of *bap* gene; (**b**) product of approximately 900 bp size in various isolates, indicative of the presence of *bla*_PER_ gene and not present in isolates A2 and A36; (**c**) product of 168 bp (presence of *csu*) in isolates A1 and A3 to A16 (not present in isolate A2); (**d**) product of approximately 578 bp (presence of *omp*A) in isolates A1–A16. PC: Positive Control, NC: Negative Control.

All the investigated genes were detected at high rates in the isolates included in this study. In particular, *bap*, *bla*_PER_, *csu*, and *omp*A were detected in 100%, 65.9%, 97.6%, and 95.1% of the strains, respectively (Figure 2). Detailed data for each isolate are presented in Table S1. The number of isolates per group of detected genes per biofilm-production class is presented in Table 7.

All four genes were identified in the majority of strong biofilm producers (21/23; 91.3%). In moderate producers, approximately half of the strains (*n* = 5; 45.5%) carried four genes, while the rest carried three genes (*n* = 5), and one strain (A32) carried only two genes. In contrast, all weak biofilm producers carried at most three genes, and only one of them carried *bla*_PER_. Hence, as indicated in Table 8, *bla*_PER_ was the gene most related to the biofilm formation status of the strains, with a statistically significant difference (Fischer’s exact test, *p* < 0.001).

## 4. Discussion

The results of this study indicate the distribution of biofilm-producing *Ab* isolates among pets in Greece, their possible dissemination in veterinary facilities, and their potential to act as challenging hospital-acquired pathogens for companion animals. To our knowledge, this is the first comprehensive investigation of biofilm formation by *Ab* isolates of animal origin in the country.

The emergence of multidrug resistance in *Acinetobacter* spp. isolates obtained from companion animals has been highlighted in numerous recent studies [11,13,15,21,22,23,24]. Nevertheless, data regarding the involvement of biofilm formation in pathogenicity and virulence in canine or feline hosts are limited. All isolates included in this study were pathogenic (obtained from infected animals) and capable of producing biofilms. This ability is considered the most important virulence factor for human strains, as it is implicated in tissue attachment and affects the intensity of the host’s immune responses [8]. In wounds, biofilms can act as physical barriers that prevent healing [25]. Dogs and cats are undoubtedly susceptible to skin and soft tissue infections (SSTIs), and a considerable percentage of samples in this study were obtained from relevant cases. Concludingly, an indication is provided that the acquisition and expression of biofilm production mechanisms enhances the ability of *Ab* to cause infections in dogs and cats, especially in cases of preceding tissue damage.

From the results of the AST and TCP tests (Table S1, Table 3, Table 5, and Table 6), a strong correlation was identified between antibiotic resistance and the production of relatively weaker biofilms. In the literature, data on the association between antibiotic resistance and biofilm production are contradictory. Perez et al. described an inverse relationship between carbapenem resistance and biofilm formation [26], which is in accordance with our results. Similar results have been reported in another study [27]. Moreover, in another study, stronger biofilm production was documented in soil bacteria in contrast to multidrug resistance [28]. However, an association between antibiotic resistance and stronger biofilm production has also been identified in other cases [2,20,29,30].

Based on the aforementioned results, the investigated isolates can be classified into two main groups (with a few exceptions). A group of multidrug-resistant and weak or moderate biofilm-producing strains, and a group of strong biofilm producers with a relatively susceptible AST profile. It is speculated that bacteria in the first group persisted in healthcare facilities and spread among animal hosts, mostly through their resistance mechanisms, since limited treatment alternatives are available in veterinary medicine (Table S1). On the other hand, bacteria in the second group were possibly distributed through their solid adherence to both abiotic and abiotic surfaces and the formation of a dense biofilm. In case of infection, this biofilm could protect the bacterial population from antimicrobial agents and implicate the outcome of the treatment, even when administered in the susceptible range [11,31]. Moreover, although these strains demonstrate a relatively susceptible profile, they are intrinsically resistant to several widely used antibiotics (ampicillin, amoxicillin–clavulanate, clindamycin, etc.). Under these circumstances, the possibility that they could acquire new resistance mechanisms due to the pressure of antibiotic exposure is considerable [11]. Therefore, the risk of the emergence of strains combining strong biofilm production and multidrug resistance in the future is definitely non-negligible.

Finally, a significant correlation between the phenotype and the area of origin was documented, since strains from Athens were regularly classified as strong biofilm producers, whereas those from Thessaloniki were as weak or moderate, regardless of the veterinary clinic in which the relevant samples were obtained. Antibiotic resistance was conversely distributed. This could indicate the spread and circulation of specific phenotypes among animal populations in each geographical region. However, the number of isolates was too limited to obtain safe conclusions.

In the molecular investigation, all the biofilm-related genes were detected at high rates. This is in accordance with recent studies examining human isolates [2,32,33]. These results confirm the positivity documented in the phenotypic test (TCP) and highlight the significance of biofilm production in the ability of *Ab* to infect canine and feline hosts, since all bacteria carried at least two of the associated genes.

Specific aspects of the genotype–phenotype correlation should be mentioned. In the majority of weak and moderate biofilm-producing strains, *bla*_PER_ was not detected, whereas it was commonly present in strong producers. The adhesion of *Ab* to plastic surfaces is associated with the presence and expression of this gene [5,11,34], and consequently, the results of the TCP test could be affected. In contrast, *bap, csu,* and *omp*A were detected in almost all isolates, regardless of their phenotypic categorization. This could be explained by variations in the expression levels of these genes, as highlighted in a recent study that included isolates of human origin [27]. In addition, the implication of variable molecular factors in the regulation of these genes [35] and the development of a mature biofilm [36] has been documented in the literature. The variance detected in the phenotypic test could be explained by the presence or absence of blaPER and the expression levels of *bap*, *csu*, and *omp*A.

This study has certain limitations. Although the examined bacteria were obtained from clinical samples, data regarding each patient’s history and medication were not available. Therefore, possible predisposing factors, severity of each infection, and treatment outcomes were not evaluated. Regarding the phenotypic assay, the quantification data provided by the TCP technique involve a level of uncertainty and co-evaluation of a direct quantification method such as counting of colony forming units (CFUs) would provide more reliable results. Moreover, only the presence of four biofilm-associated genes was examined, while the development of a mature biofilm is a complex process that is affected by numerous genes, transcription factors, and environmental conditions. Thus, the response of the mechanisms of each strain in vivo cannot be precisely predicted. The expression of the investigated genes in planktonic cells and in biofilm conditions could provide critical information and is of major importance for a circumstantial molecular interpretation. However, the rate of strong biofilm-producing strains and the presence of related genes in the investigated group highlight the importance of this procedure for the pathogenicity of *Ab* against pet hosts and its contribution to the dissemination of relevant epidemic strains among companion animals.

In reference to future perspectives, extensive epidemiological research is essential to comprehensively investigate the distribution of *Ab* strains in companion animals, their clonality, predisposing factors of their spreading, and their association with human isolates. In this regard, information regarding the history of each patient and the outcome of the administered treatment should be evaluated to provide sufficient knowledge about the management of the respective cases. Finally, environmental sampling and eradication measures in veterinary clinics should be encouraged to prevent further dissemination of relevant strains and to promote alertness among veterinarians.

## 5. Conclusions

*Acinetobacter* spp. are emerging pathogens in companion animals, especially in hospitalized animals. In Greece, *Ab* strains have been regularly isolated from infected pets over the last few years. A significant percentage of these bacteria exhibit MDR or XDR susceptibility profiles, all of which are capable of producing biofilms. Biofilm-associated genes are widely distributed among these strains. An inverse relationship between antibiotic resistance and biofilm production has been identified. Thus, the spread and persistence of *Ab* in veterinary facilities and the infection of canine and feline hosts could be accomplished through multidrug resistance, formation of mature biofilms, or both. Furthermore, the danger that companion animals could act as reservoirs of relevant epidemic strains and enhance their dissemination in the community is significant. Therefore, proper surveillance and control measures should be established, and further research should be conducted.

## Figures and Tables

**Table 1:** Classification criteria for biofilm production ability of the isolates [19,20].

Mean OD	Biofilm Production
≤ODc	No (−)
ODc < OD ≤ 2ODc	Weak (+)
2ODc < OD ≤ 4ODc	Moderate (++)
4ODc < OD	Strong (+++)

**Table 2:** Primers used in this study [2].

Primers	Primer Sequence (5′-3′)	Product Size (bp)
*bap*	TGCTGACAGTGACGTAGAACCACA	184
	TGCAACTAGTGGAATAGCAGCCCA	
*bla*_PER-1_	GCAACTGCTGCAATACTCGG	900
	ATGTGCGACCACAGTACCAG	
*csu*E	CATCTTCTATTTCGGTCCC	168
	CGGTCTGAGCATTGGTAA	
*omp*A	GTTAAAGGCGACGTAGACG	578
	CCAGTGTTATCTGTGTGACC	

**Table 3:** Absorbance data from the TCP method and respective classification.

Strain Code	Origin	Sample	1st Well	2nd Well	3rd Well	Mean Absorbance (±CI95%)	Classification of Biofilm Production
A1	Canine	Soft tissue	223	245	262	243.3 ± 18.1	moderate
A2	Canine	Pleural effusion	536	618	652	602 ± 55.1	strong
A3	Canine	Soft tissue	142	195	198	178.3 ± 16.3	weak
A4	Feline	Soft tissue	294	159	182	211.7 ± 66.7	weak
A5	Feline	Urine	259	262	231	250.7 ± 15.8	moderate
A6	Feline	Urine	232	257	332	273.7 ± 48.1	moderate
A7	Canine	Soft tissue	316	401	321	346 ± 12.7	moderate
A8	Canine	Soft tissue	212	252	224	229.3 ± 19.0	moderate
A9	Canine	Soft tissue	777	689	624	696.7 ± 71.0	strong
A10	Canine	Soft tissue	652	602	762	672 ± 75.6	strong
A11	Canine	Soft tissue	349	292	437	359.3 ± 67.5	moderate
A12	Feline	Nasal cavity	1354	1145	1247	1248.7 ± 96.6	strong
A13	Canine	Soft tissue	237	183	194	204.7 ± 26.4	weak
A14	Feline	Urine	169	182	221	190.7 ± 25.0	weak
A15	Canine	Soft tissue	242	277	329	282.7 ± 40.4	moderate
A16	Feline	Soft tissue	779	558	652	663 ± 102.5	strong
A17	Canine	Pleural effusion	683	692	643	672.7 ± 24.1	strong
A18	Feline	Soft tissue	685	616	745	682 ± 59.6	strong
A19	Canine	Soft tissue	1340	1454	1347	1380.3 ± 59.0	strong
A20	Feline	Urine	488	505	518	503.7 ± 13.9	strong
A21	Canine	Urine	1312	1156	1209	1225.7 ± 73.3	strong
A22	Feline	Urine	237	168	177	194 ± 34.7	weak
A23	Feline	Urine	589	484	500	524.3 ± 52.3	strong
A24	Feline	Nasal cavity	723	579	725	675.7 ± 77.4	strong
A25	Canine	Ear canal	701	562	724	662.3 ± 81.0	strong
A26	Canine	Urine	853	981	826	886.7 ± 76.5	strong
A27	Feline	Ear canal	646	634	512	597.3 ± 68.5	strong
A28	Canine	Ear canal	488	553	534	525 ± 30.9	strong
A29	Feline	Urine	2872	2513	2627	2670.7 ± 169.5	strong
A30	Canine	Ear canal	1363	1452	1526	1447 ± 75.4	strong
A31	Canine	Soft tissue	117	144	134	131.7 ± 12.6	weak
A32	Canine	Soft tissue	334	336	241	303.7 ± 50.2	moderate
A33	Canine	Soft tissue	451	597	412	486.7 ± 90.1	strong
A34	Canine	Soft tissue	192	202	234	209.3 ± 20.3	weak
A35	Canine	Urine	545	428	456	476.3 ± 56.5	strong
A36	Canine	Soft tissue	1384	1393	1713	1496.7 ± 173.2	strong
A37	Canine	Urine	236	239	214	229.7 ± 12.6	moderate
A38	Feline	Soft tissue	629	809	564	667.3 ± 117.3	strong
A39	Feline	Urine	252	229	237	239.3 ± 10.8	moderate
A40	Canine	Soft tissue	303	256	350	303 ± 43.4	moderate
A41	Canine	Blood	862	1042	1173	1025.7 ± 144.3	strong

**Table 4:** Isolate’s origin per category of biofilm production.

Sample Type	Number of Isolates Per Category			Total
	Strong	Moderate	Weak	
Canine	14	8	4	26
Feline	9	3	3	15
Total	23	11	7	41
Soft tissue	8	7	5	20
Urine	6	4	2	12
Ear canal	4	-	-	4
Nasal cavity	2	-	-	2
Pleural effusion	2	-	-	2
Blood	1	-	-	1
Total	23	11	7	41

**Table 5:** Antibiotic resistance rates of the isolates per category of biofilm production by the TCP method.

Antibacterial Agent	Resistance Rate % (n)				Fischer’s Exact *p*-Value
	Total	Biofilm Formation			
		Strong	Moderate	Weak	
Ampicillin + sulbactam	48.8% (20)	13% (3)	100% (11)	85.7% (6)	*p* < 0.001
Piperacillin + tazobactam	48.8% (20)	13% (3)	100% (11)	85.7% (6)	*p* < 0.001
Ceftazidime	51.2% (21)	13% (3)	100% (11)	100% (7)	*p* < 0.001
Cefepime	51.2% (21)	13% (3)	100% (11)	100% (7)	*p* < 0.001
Imipenem	48.8% (20)	13% (3)	100% (11)	85.7% (6)	*p* < 0.001
Amikacin	43.9% (18)	8.7% (2)	90.9% (10)	85.7% (6)	*p* < 0.001
Gentamicin	75.6% (31)	60.9% (14)	100% (11)	85.7% (6)	*p* = 0.03
Tobramycin	41.4% (17)	4.3% (1)	90.9% (10)	85.7% (6)	*p* < 0.001
Ciprofloxacin	100% (41)	100% (23)	100% (11)	100% (7)	*p* = 1
Enrofloxacin	100% (41)	100% (23)	100% (11)	100% (7)	*p* = 1
Sulph/zole + Trimethoprim	63.4% (26)	34.8% (8)	100% (11)	100% (7)	*p* < 0.001
Doxycycline	68.3% (28)	43.5% (10)	100% (11)	100% (7)	*p* < 0.001
Minocycline	12.2% (5)	4.3% (1)	9.1% (1)	42.9% (3)	*p* = 0.046
Total	41	23	11	7	

**Table 6:** Mean absorbance in the TCP method per result of the AST for each antibiotic.

Antibacterial Agent ^1^	Mean Absorbance (±CI95%)		*t*-Test Comparison of Independent Means *p*-Value
	Non-Resistant ^2^ Isolates	Resistant Isolates	
Ampicillin + sulbactam	879.8 ± 130.1	319.6 ± 58.1	*p* < 0.001
Piperacillin + tazobactam	879.8 ± 130.1	319.6 ± 58.1	*p* < 0.001
Ceftazidime	914.1 ± 130.7	313.7 ± 55.7	*p* < 0.001
Cefepime	914.1 ± 130.7	313.7 ± 55.7	*p* < 0.001
Imipenem	879.8 ± 130.1	319.6 ± 58.1	*p* < 0.001
Amikacin	867.1 ± 124.0	273.7 ± 27.6	*p* < 0.001
Gentamicin	802.8 ± 118.3	543.3 ± 106.4	*p* = 0.006
Tobramycin	851.2 ± 120.2	261.2 ± 24.7	*p* < 0.001
Sulph/zole + Trimethoprim	939.7 ± 162.6	414.4 ± 73.2	*p* < 0.001
Doxycycline	883.5 ± 106.7	463.0 ± 108.7	*p* < 0.001
Minocycline	654.0 ± 96.1	265.1 ± 67.0	*p* = 0.002

^1^ Enrofloxacin and Ciprofloxacin are not included since the resistance rate for these agents was 100%. ^2^ Intermediate or susceptible.

**Table 7:** Distribution of isolates per group of detected genes and class of biofilm production (TCP).

Detected Genes	Number of Isolates/Phenotypic Biofilm Formation			Total
	Strong	Moderate	Weak	
*bap*, *bla*_PER_, *csu*, *omp*A	21	5	0	26
*bap*, *csu*, *omp*A	1	5	6	12
*bap*, *bla*_PER_, *csu*	0	0	1	1
*bap*, *csu*	0	1	0	1
*bap*, *omp*A	1	0	0	1

**Table 8:** Correlation of biofilm-related genes and phenotypic biofilm formation.

	Strong	Moderate	Weak	Fischer’s Exact *p*-Value
*bap*	23/23	11/11	7/7	*p* = 1
*bla*_PER_	21/23	5/11	1/7	*p* < 0.001
*csu*	22/23	11/11	7/7	*p* = 1
*omp*A	23/23	10/11	6/7	*p* = 0.18

## Data Availability

The data presented in this study are available in the article.

## Supplementary Materials

The following supporting information can be downloaded at: https://www.mdpi.com/article/10.3390/tropicalmed9050109/s1; Table S1: Detailed data of the *Acinetobacter baumannii* strains included in this study.
